# Contrast-Enhanced Ultrasound to Monitor Early Recurrence of Primary Hepatocellular Carcinoma after Curative Treatment

**DOI:** 10.1155/2018/8910562

**Published:** 2018-11-07

**Authors:** Lian-Feng Liu, Zhan-Ling Ding, Jian-Hong Zhong, Hong-Xue Li, Jun-Jie Liu, Hang Li, Le-Qun Li

**Affiliations:** ^1^Department of Ultrasound, Affiliated Tumor Hospital of Guangxi Medical University, Nanning 530021, China; ^2^Department of Hepatobiliary Surgery, Affiliated Tumor Hospital of Guangxi Medical University, Nanning 530021, China

## Abstract

**Objective:**

To evaluate contrast-enhanced ultrasound (CEUS) for monitoring early intrahepatic recurrence of primary hepatocellular carcinoma (HCC) after curative treatment.

**Methods:**

We prospectively analyzed 97 patients (124 nodules) with primary HCC who underwent hepatic resection or radiofrequency ablation and subsequently experienced intrahepatic recurrence. Patients were assessed with conventional ultrasound and CEUS. They were also assessed with contrast-enhanced computed tomography (CECT) and/or magnetic resonance imaging (MRI). The image characteristics of CEUS of recurrent hepatocellular carcinoma and high-grade dysplastic nodules (HGDNs) were analyzed. In addition, the ability of CEUS and CECT/MRI to assess internal artery vascularization in recurrent disease was compared.

**Results:**

CEUS of recurrent hepatocellular carcinoma showed hyperenhancement in the arterial phase in 96 of 99 nodules, and it showed hypo- or isoenhancement for portal venous and delayed phases. The most common enhancement patterns were “fast-in and slow-out” and “fast-in and fast-out”. Based on the arterial hyperenhancement of lesions and with clinical data such as patient history of HCC and increased level of serum alpha-fetoprotein, the diagnostic accuracy of CEUS for recurrent HCC was significantly higher than that based on the enhancement pattern of “fast-in and fast-out”. CEUS of HGDNs showed local or global hyperenhancement during the arterial phase, isoenhancement during the portal venous phase, and isoenhancement or slight hypoenhancement during the delayed phase. The enhancement pattern was “fast-in and slow-out”. In some cases, it was difficult to differentiate HGDNs from recurrent disease using CEUS. Vascularization in recurrent disease was significantly higher when assessed by CEUS than when assessed with CECT/MRI (*P* < 0.05). For detecting recurrent disease, CEUS showed sensitivity of 97.0%, specificity of 68.0%, positive predictive value of 92.3%, and negative predictive value of 85.0%. The corresponding parameters for CECT/MRI were 71.7%, 72.0%, 88.8%, and 39.1%.

**Conclusion:**

Intrahepatic recurrent HCC and HGDNs with diameter ≤ 3.0 cm have a characteristic appearance on CEUS. This imaging modality may be effective for monitoring early intrahepatic recurrence after curative treatment of primary HCC.

## 1. Introduction

Globally hepatocellular carcinoma (HCC) ranks sixth in incidence among all cancers, and it is the second leading cause of cancer-related deaths [1]. The long-term overall survival is poor even after curative resection or ablation because of the high recurrence rate, which can reach 74% within 5 years after curative treatment [2, 3]. In fact, the 5-year recurrence rate is approximately 25% among patients who undergo liver transplantation [4]. Therefore, early detection and diagnosis of postoperative intrahepatic lesions are an important strategy to further improve the efficacy of curative resection/ablation and improve patients' long-term prognosis [5].

Effective early detection likely requires the ability to identify dysplastic nodules, which are considered a precancerous HCC lesion. Such nodules can be classified as low or high grade, and malignant progression of high-grade dysplastic nodules (HGDNs) appears to cause most cases of HCC[6, 7]. In patients with HGDNs, HCC subsequently occurs in 38% within 1 year, 41% within 2 years, 51% within 3 years, and 51% within 4 years [8].

Recurrent HCC and dysplastic nodules are currently diagnosed based on imaging with ultrasound, contrast-enhanced computed tomography (CECT), and magnetic resonance imaging (MRI). Conventional ultrasound is fast, simple, and inexpensive and does not involve radiation, making it the preferred method to monitor HCC patients after curative resection [9]. However, it can be poor at displaying blood perfusion in lesions, especially in patients with cirrhosis. Computed tomography and MRI can easily fail to detect, or lead to misdiagnosis of, small lesions and lesions showing early enhancement.

A superior alternative for monitoring HCC recurrence may be contrast-enhanced ultrasound (CEUS), which has greatly improved our ability to detect and differentially diagnose small intrahepatic lesions. In contrast to CECT/MRI, CEUS allows endovascular imaging and real-time imaging of blood perfusion [10] using a relatively simple device and procedures. Despite the strong clinical abilities of CEUS, few studies have examined its usefulness for HCC monitoring after curative therapy. Therefore. The present study analyzed CEUS imaging of intrahepatic recurrent HCC and HGDNs with a diameter < 3 cm, and it assessed the ability of CEUS to detect arterial vascularization in recurrent lesions in comparison to CECT/MRI.

## 2. Methods

### 2.1. Patients

From February 2015 to March 2017, a total of 369 patients at our hospital (340 men, 29 women; mean age, 52.7±11.1 years) were followed up once every 3 or 6 months by conventional ultrasound after hepatic resection or radiofrequency ablation of primary HCC. Follow-up time ranged from 1 to 3.2 years (median, 2.6 years). From this cohort of patients, those satisfying the following four criteria were prospectively analyzed in the present study: (1) fewer than 4 new suspicious nodules (diameter ≤ 3.0 cm) discovered on follow-up; (2) no extrahepatic metastatic lesions; (3) new suspicious nodules examined by CEUS, CECT, and/or MRI; and (4) nodules confirmed by pathology. The study was approved by the Ethics Committee of the Affiliated Tumor Hospital of Guangxi Medical University, and all patients provided written informed consent prior to clinical examinations.

### 2.2. CEUS Imaging

Prior to CEUS, conventional ultrasound was performed to determine lesion location, quantity, size, formation, internal echo, blood supply, and lesion relationship to peripheral structure. If lesions could not be seen clearly, then they were located using other modalities.

CEUS was performed using the color Doppler LOGIQ E9 instrument (GE) with a transducer frequency of 2.0-5.0 Hz and a mechanical index of 0.12-0.18. The contrast agent was sulphur hexafluoride microbubbles (SonoVue, Bracco, Italy). The microbubbles were prepared by dissolving 24.98 mg dry powder in 5 mL NaCl saline to a final concentration of 8 *μ*L/mL. For each set of images, 2.4 mL of contrast agent suspension was injected within 3-5 sec through a superficial venous indwelling needle preinserted into the elbow, followed by a flush of 5 mL saline. While the contrast agent was injected, the timing device was started and dynamic images were recorded. Enhancement of the lesions and peripheral hepatic tissue were observed over 4-6 minutes, and the procedure was repeated if the results were unsatisfactory. Multiple injections of contrast agent were made at intervals of at least 15 min.

### 2.3. Interpretation of CEUS Images

After CEUS, two experienced sonographers played back the video and determined the degree and pattern of enhancement of those lesions in different phases based on the guidelines of the European Union of Medical and Biological Ultrasound Societies [11]. The following CEUS phases were defined: arterial (10-30 sec), portal venous (31-120 sec), and delayed (121-360 sec). Differences between the assessments were resolved through discussion.

Enhancement of the lesion was classified as greater than (hyperenhancement), equal to (isoenhancement), or less than (hypoenhancement) the enhancement of the peripheral hepatic parenchyma at the same depth as the reference. If contrast agent did not enter the lesion, the result was classified as nonenhancement.

Patterns of enhancement were assigned to one of the following: (1)* fast-in and fast-out*, when lesions were hyperenhanced in the arterial phase and hypoenhanced in the portal venous and delayed phases; (2)* fast-in and slow-out*, when lesions were hyperenhanced in the arterial phase, iso- or hyperenhanced in the portal venous phase, and hypoenhanced in the delayed phase; (3)* slow-in and fast-out*, when enhancement occurred in the late arterial and early portal venous phases, followed by regression in the later portal venous phase; (4)* slow-in and slow-out,* when lesions were enhanced in the late arterial and portal venous phases, with regression or no regression in the delayed phase; (5)* sync-in and sync-out*, when lesions were isoenhanced in all three phases; and (6)* slow-in and sync-out*, when lesions were hypoenhanced in the arterial phase and isoenhanced in the portal venous and delayed phases.

### 2.4. Statistical Analysis

Results were reported as mean ± SD and analyzed using SPSS 19.0 (IBM, Chicago, IL, USA). Differences were assessed for significance using the chi-squared test. P < 0.05 was defined as significant.

## 3. Results

In this study, 124 liver nodules measuring 6-30 mm were analyzed in 97 patients, of whom 75 had one nodule, 17 patients had two nodules, and 5 patients had three nodules. Of the nodules, 99 were determined by pathology to be recurrent HCC, 8 to be HGDNs, and 17 to be low-grade dysplastic nodules or regenerated nodules (RN).

### 3.1. Features of Lesion Enhancement by CEUS

**Figure 1:**
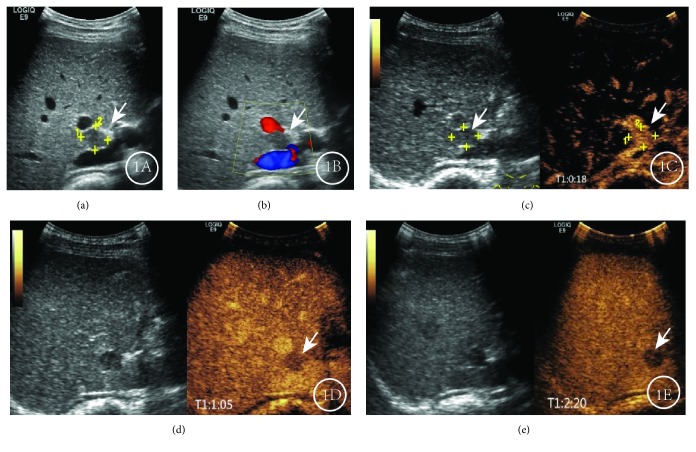
Results for a male patient, aged 46, in whom HCC recurred at 6 months after curative surgery. (a) Routine ultrasound showed hypoechoic nodules (1.8 × 1.5 cm) in the right anterior lobe near the secondary porta of liver. (b) Color Doppler flow imaging (CDFI) showed no color flow signal around or inside the nodule. (c) CEUS showed rapid, uniform enhancement in the arterial phase. (d) CEUS showed a low, rapidly decreasing enhancement in the portal phase. (e) CEUS showed low enhancement in the delayed phase. This CEUS expression pattern was “fast-in, fast-out”.

**Figure 2:**
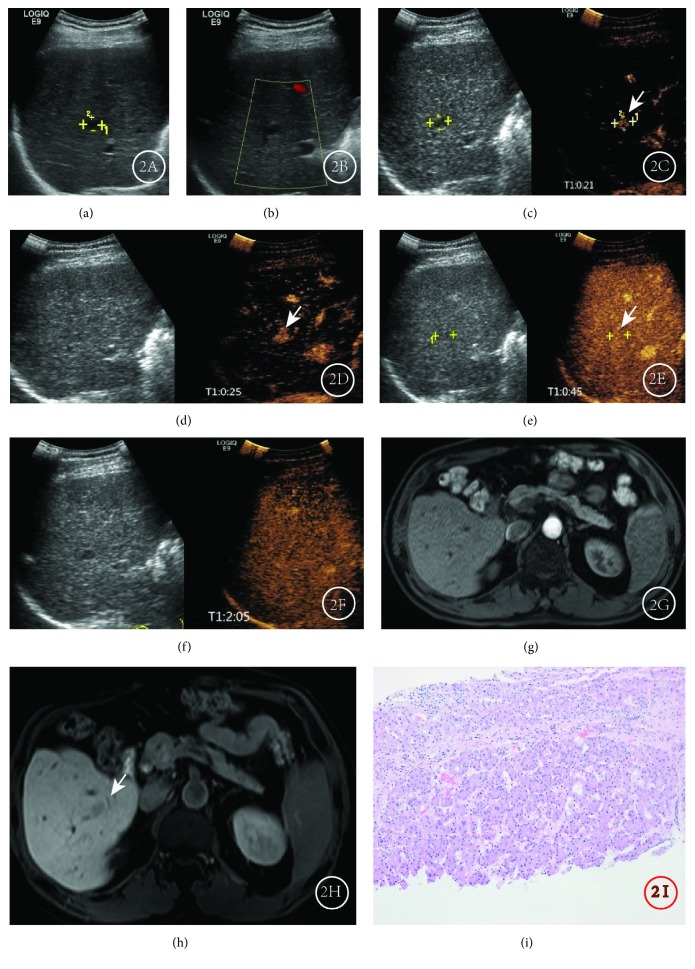
Results for a male patient, aged 41, in whom HCC recurred at 1 year after surgery. (a) Conventional ultrasound showed a low echogenic nodule (0.9 × 1.2 cm) in the lower lobe of the right liver. (b) CDFI showed no color flow signal around or inside the nodule. (c, d) CEUS showed rapid, uniform enhancement in the arterial phase. (e, f) CEUS showed isoenhancement during the portal vein and delayed phases. This CEUS expression pattern was “fast-in, slow-out”. (g) MRI revealed no abnormal enhancement lesions in the arterial phase. (h) MRI showed a slightly lower signal nodule in the lower right segment of the liver in the hepatobiliary phase, and the nodules showed no difference from MRI performed 3 months before. Nodules were suspected of being liver cirrhosis. (i) Hematoxylin-eosin staining of pathology biopsy revealed highly differentiated HCC.

**Figure 3:**
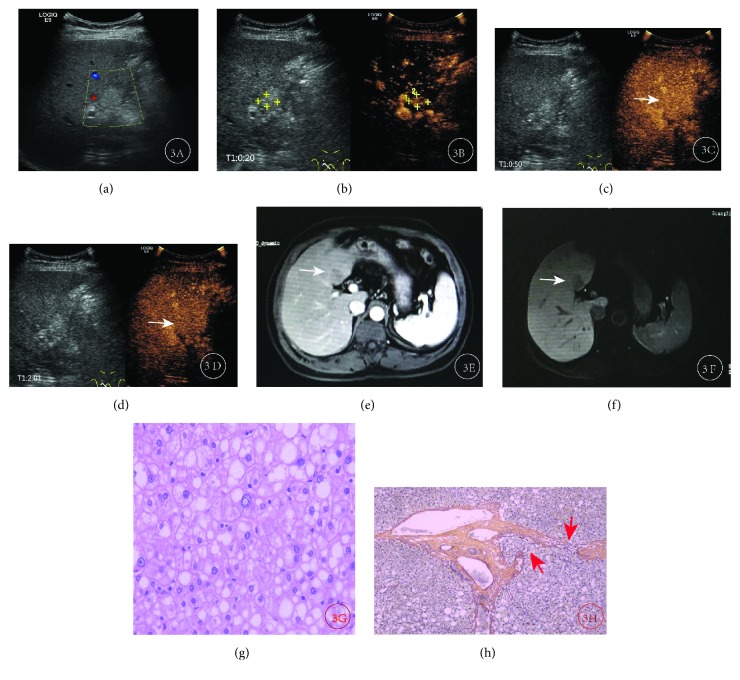
Results for a male patient, aged 55, in whom recurrence occurred 11 years after surgery. A lesion persisted near the first hepatic portal for more than 5 years, and more recent examination suggested that it was growing. The lesion was negative for alpha-fetoprotein. (a) Conventional ultrasound revealed a slightly hyperecho lesion (2.8 × 2.6 cm) in the right lobe near the first hepatic portal area. No significant color flow signal was seen by CDFI. (b) CEUS showed rapid, strong enhancement in the arterial phase, with a range of approximately 1.2 × 1.0 cm. (c, d) CEUS showed isoenhancement in the portal and delayed phases. (e, f) MRI showed no obvious enhancement in the arterial phase and low signal in the hepatobiliary phase. (g) Hematoxylin-eosin staining of pathology biopsy showed many lipid droplets in liver cells and heterogeneous hyperplasia. The ratio of dual nuclear and nuclear plasma was elevated, and nuclei contained inclusion bodies. (f) Pathology staining for reticular fibers showed that the portal area was partially invaded, so the lesion was diagnosed as HGDN.

Taking the degree of enhancement of hepatic parenchyma as the reference, In the 99 recurrent HCC lesions, we found that 96 recurrent HCC lesions were hyperenhanced in the arterial phase, accounting for 97.0% (96/99), and hypo- or isoenhanced in the portal venous and delayed phases. Eight HGDNs were locally or globally hyperenhanced in the arterial phase, isoenhanced in the portal venous phase, and isoenhanced or slightly hypoenhanced in the delayed phase. A total of 17 regenerated nodules or low-grade dysplastic nodules were hypo- or isoenhanced in the arterial phase and isoenhanced in the portal venous and delayed phases (Table 1). Of the 99 recurrent HCC lesions, 36 showed an enhancement pattern of “fast-in and fast-out” (Figure 1), 60 showed a pattern of “fast-in and slow-out” (Figure 2), and 3 showed a pattern of “slow-in and fast-out”. Eight HGDNs showed a pattern of “fast-in and slow-out” (Figure 3), while all 17 regenerated nodules or low-grade dysplastic nodules showed a pattern of “sync-in and sync-out” or “slow-in and sync-out” (Table 2).

The enhancement pattern of “fast-in and fast-out” showed a diagnostic accuracy of 36.4% (36/99) on its own, and this increased to 97.0% (96/99) if this pattern was combined with a “fast-in” observation of arterial hyperenhancement and with clinical data such as patient history of HCC and increased level of serum alpha-fetoprotein, with a sensitivity of 97.0%, specificity of 68.0%, positive predicative value (PPV) of 95.1%, and negative predicative value (NPV) of 85.0%.

### 3.2. Comparison of CEUS and CECT/MRI for Diagnosing Recurrent HCC

Among the 99 recurrent HCC lesions, 61 showed a typical “fast-in and fast-out” pattern by CECT/MRI, which was significantly lower than the 97.0% accuracy based on a CEUS pattern of “fast-in” in the arterial phase. A total of 38 of 99 recurrent HCC lesions showed atypical manifestations, were never diagnosed, or were diagnosed with a delay. Of these 38 lesions, 35 (92.1%) were hyperenhanced in the arterial phase by CEUS (Table 3).

## 4. Discussion

HCC shows a high degree of malignancy and strong potential for invasion and metastasis, which counteract the efficacy of available therapies [12, 13]. Therefore, early detection, diagnosis, and treatment of intrahepatic recurrent tumors are an important way to improve therapeutic effects and extend patients' lives [14]. Conventional ultrasound, CECT, and MRI are not ideal for this task because they do not show high specificity for detecting the HGDNs that give rise to HCC [15]. Here we provide evidence that CEUS may perform better than these other modalities for detecting HGDNs and therefore recurrent HCCs.

Recurrent HCC seems to arise through intrahepatic metastasis of initial lesions or through nodular sclerosis with malignancy [16]. The development of nodular sclerosis into HCC is a gradual process with multiple phases [17], and conventional ultrasound, CECT, or MRI cannot differentiate the two conditions well. CEUS may perform better because it can detect the change in blood supply from the portal vein to the hepatic artery during progression to HCC. In our study, 8 HGDNs showed a CEUS enhancement pattern of “fast-in and slow-out”: during the arterial phase, 6 lesions were globally slightly enhanced or hyperenhanced, while 2 lesions were locally hyperenhanced. During the portal venous phase, all 8 lesions were isoenhanced, while they were iso- or hypoenhanced during the delayed phase.

CEUS may not differentiate HGDNs from well-differentiated small HCC, which is another step en route from nodular sclerosis to HCC. However, this is less clinically relevant, because active intervention is required in either case. Changes in the blood supply of HGDNs, mainly from the portal vein to the hepatic artery, are dangerous signs of malignant transformation [18] and should receive active intervention.

CEUS gives different results depending on whether the tissue is recurrent or primary HCC [19]. In the case of recurrent HCC, the arterial phase enhancement is slower in contrast agent remission and may be consistently enhanced in the portal or delayed phases; as a result, the enhancement pattern “fast forward and slow-out” occurs more often in recurrent than primary disease. One study suggests that the pattern of typical “fast-in and fast-out” only accounts for 40% for recurrent HCC; the enhancement pattern of “fast-in and slow-out” also has a relatively higher positive predictive value, which is 97% for recurrent HCC [19]. Those authors concluded that lesions showing a "fast-in and slow-out" enhancement pattern are more likely to recur. Our results are consistent with that possibility: the CEUS enhancement pattern of “fast-in and fast-out” predicted 36 of 99 recurrent lesions, but the pattern of “fast-in and slow-out” predicted 60. These results may reflect that imaging findings for smaller tumors are less specific because of incomplete neovascularization [20] and that regression time of contrast agent in the portal and delayed phases is longer for well-differentiated than for moderately or poorly differentiated liver cancer [21]. These results may also reflect that if recurrence occurs at a single site through intrahepatic metastasis of primary HCC, the imaging findings should be similar to those of primary disease, whereas the enhancement would be quite different if the recurrence occurred at multiple sites through the malignant transformation of hardened nodules [11, 22]. During the transformation from dysplastic nodules to early HCC or from well-differentiated to poorly differentiated HCC [23], blood supply to the lesion changes, with arterial blood supply gradually increasing and portal vein blood supply decreasing within the lesion.

Our results suggest that, in patients with a history of HCC, lesions showing CEUS enhancement patterns of “fast-in and fast-out” or “fast-in and slow-out” should be considered at greater risk of recurrence. At the same time, diagnostic accuracy for recurrence was only 36.4% when based only on the pattern of “fast-in and fast-out”. This rate increased to 97.0% when diagnosis took into account arterial “fast-in” enhancement, and sensitivity and positive predictive value were also high in this case. Our observation of three cases of recurrent HCC with a pattern of “slow-in and fast-out” suggests that the possibility of malignancy should not be ruled out as long as regression occurs during the portal venous and delayed phases. Further work should examine how typical CEUS findings for recurrent HCC depend on treatment history, such as resection or ablation. Changes in local blood perfusion after treatment affect the time of contrast agent perfusion.

Dynamic monitoring of blood supply within the lesion and early detection of arterial vascularization is one of the most important methods for early diagnosis of HCC [24]. Decreased portal vein blood supply and increased arterial blood supply are important indicators of HCC formation, and they are typically assessed using CECT/MRI. This approach, however, can give inaccurate results with small lesions because of effects related to scanning interval, volume effect, and scanning time period. For example, one study reported that, for small lesions < 2.0 cm, high enhancement of the arterial phase appears as abnormal vascular perfusion in 70-90% of CECT/MRI analyses [25, 26]. This suggests that the arterial phase may be useful for predicting recurrence based on small lesions. In our study, it shows that CEUS was significantly better than CECT/MRI for detecting vascularization of small lesions.

## 5. Conclusions

Our relatively small study suggests that CEUS can reveal typical enhancement patterns useful for differentiating intrahepatic recurrent HCC and HGDNs of diameter ≤3.0 cm. CEUS may be useful for monitoring early intrahepatic recurrence after treatment for primary liver cancer.

## Figures and Tables

**Table 1:** CEUS enhancement of the 124 lesions in different phases.

Phase	Enhancement	Recurrent HCC (n=99)	High-grade dysplastic nodules (n=8)	regenerate nodules or low-grade dysplastic nodules (n=17)
Arterial				
	hypo	0	0	5
	iso	3	0	12
	hyper	96	8	0
Portal venous				
	hypo	39	0	0
	iso	60	8	17
	hyper	0	0	0
Delayed				
	hypo	50	2	0
	iso	49	6	17
	hyper	0	0	0

**Table 2:** Distribution of CEUS enhancement patterns for the 124 lesions.

Pattern	Recurrent HCC (n=99)	High-grade dysplastic nodules (n=8)	regenerate nodules or low-grade dysplastic nodules (n=17)
fast-in and fast-out	36 (36.4)	0	0
fast-in and slow-out	60 (60.6)	8 (100)	0
slow-in and fast-out	3 (3.0)	0	0
sync-in and sync-out or slow-in and sync-out	0	0	17 (100)

Values are n (%).

**Table 3:** Comparison of CEUS and CECT/MRI in the detection of arterial vascularization in recurrent HCC based on high enhancement in the arterial phase.

	Recurrent HCC		Sensitivity	Specificity	PPV	NPV
	Yes (n=99)	No (n=25)				
Enhancement by CEUS						
Yes	96	8	97.0% (96/99)	68.0% (17/25)	92.3% (96/104)	85.0% (17/20)
No	3	17				
Enhancement by CECT/MRI						
Yes	71	9	71.7% (71/99)	72.0% (18/25)	88.8% (71/80)	39.1% (18/46)
No	28	18				
*P*	<0.001					

PPV, positive predictive value; NPV, negative predictive value.

## Data Availability

The data used to support the findings of this study are available from the corresponding author upon request.
